# Leveraging a genetically-informative study design to explore depression as a risk factor for type 2 diabetes: Rationale and participant characteristics of the Mood and Immune Regulation in Twins Study

**DOI:** 10.3389/fcdhc.2023.1026402

**Published:** 2023-03-17

**Authors:** Briana Mezuk, Kristen Kelly, Erica Bennion, Jeannie B. Concha

**Affiliations:** ^1^ Center for Social Epidemiology and Population Health, Department of Epidemiology, University of Michigan School of Public Health, Ann Arbor, MI, United States; ^2^ Research Center for Group Dynamics, Institute for Social Research, University of Michigan, Ann Arbor, MI, United States; ^3^ Institute for Behavioral Genetics, University of Colorado Boulder, Boulder, CO, United States; ^4^ Office of Maternal and Child Health, Utah Department of Health and Human Services, Salt Lake, UT, United States; ^5^ College of Health Sciences, University of Texas at El Paso, El Paso, TX, United States

**Keywords:** depression, co-twin, gene-environment interaction, stress, allostatic load, inflammation, diabetes

## Abstract

**Background:**

Comorbidity between depression and type 2 diabetes is thought to arise from the joint effects of psychological, behavioral, and biological processes. Studies of monozygotic twins may provide a unique opportunity for clarifying how these processes inter-relate. This paper describes the rationale, characteristics, and initial findings of a longitudinal co-twin study aimed at examining the biopsychosocial mechanisms linking depression and risk of diabetes in mid-life.

**Methods:**

Participants in the Mood and Immune Regulation in Twins (MIRT) Study were recruited from the Mid-Atlantic Twin Registry. MIRT consisted of 94 individuals who do not have diabetes at baseline, representing 43 twin pairs (41 monozygotic and 2 dizygotic), one set of monozygotic triplets, and 5 individuals whose co-twin did not participate. A broad set of variables were assessed including *psychological factors* (e.g., lifetime history major depression (MD)); *social factors* (e.g., stress perceptions and experiences); and *biological factors*, including indicators of metabolic risk (e.g., BMI, blood pressure (BP), HbA1c) and immune functioning (e.g., pro- and anti-inflammatory cytokines), as well as collection of RNA. Participants were re-assessed 6-month later. Intra-class correlation coefficients (ICC) and descriptive comparisons were used to explore variation in these psychological, social, and biological factors across time and within pairs.

**Results:**

Mean age was 53 years, 68% were female, and 77% identified as white. One-third had a history of MD, and 18 sibling sets were discordant for MD. MD was associated with higher systolic (139.1 vs 132.2 mmHg, p=0.05) and diastolic BP (87.2 vs. 80.8 mmHg, p=0.002) and IL-6 (1.47 vs. 0.93 pg/mL, p=0.001). MD was not associated with BMI, HbA1c, or other immune markers. While the biological characteristics of the co-twins were significantly correlated, all within-person ICCs were higher than the within-pair correlations (e.g., HbA1c within-person ICC=0.88 vs. within-pair ICC=0.49; IL-6 within-person ICC=0.64 vs. within-pair=0.54). Among the pairs discordant for MD, depression was not substantially associated with metabolic or immune markers, but was positively associated with stress.

**Conclusions:**

Twin studies have the potential to clarify the biopsychosocial processes linking depression and diabetes, and recently completed processing of RNA samples from MIRT permits future exploration of gene expression as a potential mechanism.

## Background

Over the past three decades, population and clinic-based studies have established that depression and type 2 diabetes are linked over the lifespan, likely through psychological, behavioral, social and biological processes (1). Understanding these complex processes in a comprehensive manner requires innovative and integrative approaches to study design, data collection, and statistical analysis (2).

Genetically-informative study designs (i.e., designs that derive inferences about, or otherwise account for, genetic liability by comparing twins, siblings, or extended family members) provide a rigorous means to investigate exposures that cannot be randomized, either due to the intrinsic nature of the exposure (e.g., exposures that are likely to cause harm) or due to the long latency period thought to underlie the exposure-outcome relationship (e.g., early life psychosocial factors and health outcomes in mid and late-life) (3). These challenges are central to efforts to examine whether, how, and under what conditions psychosocial factors, such as depression, relate to the risk, prognosis, or outcomes of diabetes over the life course (4). Understanding the various ways that genetic and environmental factors interrelate – such as gene-environment interaction, gene-environment correlation, and epigenetic modification – to influence the comorbidity between depression and type 2 diabetes can inform intervention strategies within the precision health framework (5, 6).

### Rationale of the co-twin study design

One of the most widely used genetically informative study designs is the co-twin design (3). Monozygotic twins (MZ) share 100% of their genes identical by descent, are intrinsically “matched” on important individual-level confounders (e.g., sex, age, race), and are usually raised in the same household thus controlling for family-level socioeconomic and other early life factors (7). Because of this, MZ twins can be conceptualized as a naturally-occurring “counterfactual” because they essentially start life with the same set of genetic and environmental liabilities and assets, or, at the very least, are better matched on these observed – and unobserved – liabilities and assets than standard case-control studies could achieve. As a result, comparing outcomes of MZ twins provides a unique opportunity for studying how environmental exposures influence health, including questions related to the accumulation of risk and protective factors, the influence of timing of exposures, and variation in the consequences of those exposures over the life course.

Despite these conceptual strengths of the twin design, to date there have been few longitudinal twin studies focused on the relationship between depression and type 2 diabetes, particularly outside of Northern Europe (4). Existing twin studies of this comorbidity indicate little evidence of shared genetic factors between depression and diabetes (8–10), and there evidence to support a bi-directional phenotypic causation model (that is, even accounting for genetic and environmental confounders, depression is associated with increased risk of diabetes, and vice versa) (4). While population-based twin registries have the advantage of large samples, they often lack detailed assessment of psychosocial factors salient to health (e.g., mental health, stress exposure, coping, personality, health behaviors) and may also have limited biological data that could be used to identify mechanisms linking said psychosocial factors to health outcomes. Finally, being primarily limited to Northern Europe, twin registries often have limited representation of racial/ethnic minorities. In sum, there are few twin studies of middle-age and older adults that have comprehensive measurement of psychosocial factors, objective clinical assessments (i.e., biomarkers, anthropomorphic measures), and gene expression outcomes. This latter component is increasingly recognized as a mechanism by which environmental exposures become “embodied” and influence health (2, 11).

### Conceptual framework: The biopsychosocial model

First articulated by Engel (1977) (12) and now a foundational component of health psychology (2), the biopsychosocial model provides a unified framework for considering how psychological, social, and biological aspects of health inter-relate over the life course. This model rejects the reductionist notion that the causes of complex diseases (e.g., diabetes, depression) can be solely understood as a function of biological changes. Instead, it emphasizes the importance of psychological and social factors as salient to etiopathology. In terms of how psychological factors may influence risk of type 2 diabetes, prior work has shown that depressive symptoms are correlated with biological factors such as inflammation (13–15) and hypothalamic-pituitary-adrenal (HPA) axis dysregulation (16–18), which are in turn associated with diabetes risk (19). In addition, these psychological factors are also associated with health behaviors (e.g., poor diet, physical inactivity, sleep disturbances), which are established causes of diabetes. Also, the biopsychosocial model explicitly calls for research that uses a “systems” approach, i.e., considers individuals as nested within social and physical environments, as a means of understanding these multiple determinants of health in an integrated manner (12). Finally, this model has direct implications for interventions, as it suggests there may be opportunities to modify psychosocial risk factors as a means to prevent or improve management of diabetes (20, 21).

### Aims of the current study

This paper describes the rationale, design and data collection protocol, participant characteristics, and initial findings regarding psychological, social, and biological correlates of diabetes risk from the Mood and Immune Regulation in Twins (MIRT) Study, a recently completed monozygotic co-twin study recruited from the Mid-Atlantic Twin Registry (MATR). The goal of the MIRT Study was to assess whether and how depression and related psychosocial factors (e.g., stress) influence immune functioning and risk of type 2 diabetes in mid-life. This study’s detailed phenotypic and environmental measures and longitudinal biomarker and gene expression data provide a novel resource for exploring the mechanisms underlying these hypothesized etiologic relationships.

## Methods

### Sample

Recruitment for the MIRT Study was conducted through the Mid-Atlantic Twin Registry (MATR), based at Virginia Commonwealth University (VCU). The MATR is the largest twin registry in the United States (22), with approximately 300,000 twin pairs recruited primarily from Virginia, North Carolina, and South Carolina. The sampling frame for MIRT was limited to complete pairs of self-identified monozygotic (MZ) twins (see *Results* for genetic confirmation of zygosity) aged 40 to 70 (ages when incidence of type 2 diabetes is highest), who both lived within a two-hour drive of the VCU Medical Center (a catchment area which covered the most populous counties in Virginia), where all data collection took place. Participants’ parking fees were paid for by the study, and a car service was provided for those who were unable to drive themselves. Exclusion criteria included current diagnosis of diabetes (type 1 or 2), an unstable medical condition (e.g., currently receiving treatment for cancer), or use of atypical antipsychotic medications, as these are known to influence diabetes risk.

**Figure 1:**
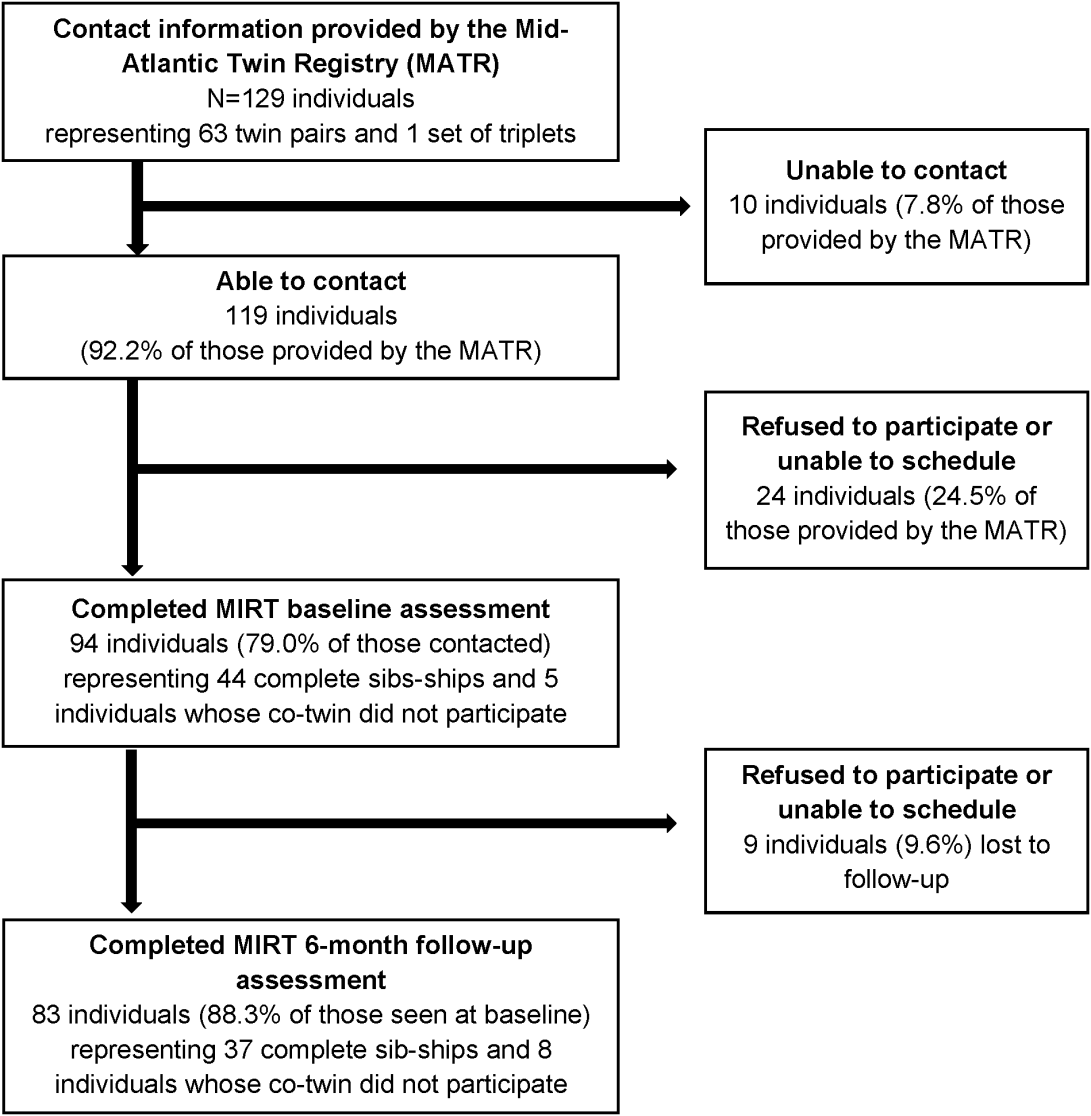
Flowchart of participant recruitment and retention in the Mood and Immune Regulation in Twins (MIRT) Study. Eligible and potentially interested twin pairs were first identified and screened by the MATR, which then provided their contact information to the MIRT Study team for recruitment. Sib-ships include twin pairs and one set of triplets. All participants believed themselves to be monozygotic at the time of study enrollment. Genetic testing was conducted as part of the baseline assessment to confirm zygosity, which determined that 42 of the 44 twin pairs and the sole set of triplets were indeed genetically identical. Genetic testing was not conducted on those individuals whose co-twin did not participate in the study, as DNA from both members is necessary to determine zygosity.

Potential participants were identified and screened for eligibility by MATR staff by telephone; individuals were told that only complete twin pairs were eligible for the study but were not told if their co-twin had agreed to be contacted by MIRT staff to preserve confidentiality. Once contacted by MATR, if both members of the pair were willing to participate, their contact information was passed on to MIRT staff who conducted recruitment into the study and scheduling. As shown by **Figure 1**, contact information for 129 individuals (representing 63 complete twin pairs and one complete set of triplets) was provided to MIRT staff by the MATR. Overall, 94 individuals (representing 44 complete sib-ships) completed the baseline assessment, and of these 83 individuals (representing 37 complete sib-ships) also completed the 6-month follow-up assessment.

The MIRT study can thus be conceptualized as a two-by-two study design, in which individuals can be compared both to their co-twin at baseline, as well as to themselves at the 6-month follow-up. The 6-month follow-up period was chosen because it provides a long-enough window of time for meaningful variation in psychosocial factors (e.g., perceived stress), and thus sets the stage for future studies to examine the stability of observed relationships between psychosocial factors with gene expression, as described in more detail in the *Discussion*.

This study was approved by the Institutional Review Board at VCU (HM15108), and all participants provided written informed consent. Participants received a report from their clinical assessment to share with their healthcare provider and received an honorarium of $125 ($50 for the baseline assessment and $75 for the follow-up) to compensate them for their time. This manuscript was pre-registered at the Open Science Framework (23), where we also share the recruitment and data collection materials and data codebooks.

### Data collection protocol

All data collection occurred at the Clinical Research Services Unit at VCU School of Medicine in Richmond, Virginia between 2014 and 2016. Participants were instructed to fast before their scheduled appointment and reported on the time and date of their last meal during the interview. After obtaining written informed consent, data collection procedures involved of two parts: first, participants underwent a clinical evaluation which included a blood draw and assessment of blood pressure (BP, measured twice using an electronic sphygmomanometer), weight and height (measured without shoes and wearing light clothing), and waist circumference (measuring using a flexible tape measure) by a trained research nurse. Afterward, participants completed an in-person interview administered by a trained research assistant. These same assessments were repeated twice: once at baseline and again approximately 6 months later. While both members of the twin pair were not required to attend the data collection procedures at the same time, many elected to do so; in all such cases, the personal interview was conducted in separate private rooms by different interviewers.

### Clinical assessment and biological measures

A 30cc venous blood draw was used to collect whole blood for extracting DNA to confirm zygosity status, for analysis of circulating levels of biomarkers of diabetes risk and immune functioning, and for collection of RNA to quantify gene expression. All participants believed themselves to be monozygotic at the time of study enrollment (only a handful had ever undergone genetic testing to confirm this belief). Zygosity testing was conducted by the Virginia Institute for Psychiatric and Behavioral Genetics (VIPBG) lab by comparing genotypes at 19 single nucleotide polymorphisms (SNPs). This comparison confirmed that all but two pairs were indeed MZ (i.e., identical alleles at all SNPs); the set of triplets was also confirmed to be monozygotic. The two pairs that were dizygotic (DZ) were retained for the descriptive statistics of the sample but excluded from the ICC analysis.

Markers of diabetes risk (i.e., hemoglobin A1c (HbA1c, an indicator of average blood glucose over the past 30-90 days), random glucose, and insulin) were analyzed by the VCU Pathology and Core Laboratory using standard laboratory protocols. Serum levels were measured of several immune-related biomarkers were analyzed by the Center for Biobehavioral Research at the VCU School of Nursing. These markers were chosen based on prior literature and to provide coverage of both pro- and anti-inflammatory processes (24). The markers included interleukin 6 (IL-6), soluble tumor necrosis factor receptor 2 (TNF-RII), high-sensitivity C-reactive protein (CRP), interleukin 10 (IL-10), interleukin 1 receptor antagonist (IL-1ra), and cortisol. R&D Systems Quantkine ELISA tests were used to measure CRP, IL-1ra, IL-6, IL-10, and TNF-RII. The R&D Systems Parameter Cortisol test was used to measure cortisol. Details of these tests can be found in **Supplementary Table 1**.

Venous blood samples for analysis of RNA were collected using Paxgene tubes and stored at VIPBG as whole blood frozen at -80 degrees Celsius until being shipped to the University of Michigan (UM), where the UM Central Biorepository performed RNA extraction. RNA was then delivered to the UM Advanced Genomics Core, where they underwent Qiagen 3’ UPX library preparation (25), a library preparation method that captures only the 3’ end of each transcript, allowing for accurate quantification of expression without transcript length bias (26). Six samples had a concentration less than 10ng and increased *via* bead purify to before UPX library preparation. This was followed by RNA sequencing on an Illumina NovaSeq 6000 S1 device (27). To eliminate the influence of batch effects on within-pair comparisons, for each participant both their baseline and 6-month follow-up samples, and those of their co-twin, were assigned to the same batch; this was done for both library preparation and RNA sequencing. This assignment reflects our prioritization of within-sibship comparisons, since within-sibship comparisons will allow future analyses to make inferences about the association between MD and gene expression in individuals matched for genotype and early-life factors.

### Personal interview and psychosocial measures

After completing the clinical assessment and blood draw with the research nurse, participants were taken into a private room where they were offered a snack and water and then underwent a personal interview administered by a trained research assistant. The interview took an average of 60 minutes to complete. It assessed a range of topics including demographic and economic characteristics, history of major depression, health-related behaviors (e.g., tobacco use, alcohol use, diet, physical activity, sleep), personality, medication use, exposure to stress, coping strategies, neighborhood characteristics, and personal and family history of health conditions. The MIRT survey instruments are provided in an Open Science repository (https://osf.io/3mxuv).

*Depression*. Lifetime history of major depression (MD) was measured at the baseline assessment only using the Diagnostic Interview Schedule (DIS) (28). The DIS is a full-structured diagnostic interview that assesses the criteria for MD according to the Diagnostic and Statistical Manual of Mental Disorders-IV (DSM-IV). These criteria include the nine symptom groups (i.e., dysphoria, anhedonia, sleeping disturbances, appetite disturbances, fatigue, concentration problems, psychomotor agitation/retardation, guilt/worthlessness, and preoccupation with death/suicidal ideation) as well as indicators of onset, recency, and impairment due to those symptoms. While there is no gold standard for measurement of MD, the DIS has moderate concordance with assessment of MD as compared to clinical psychiatric interviews (28). Although the version of the DIS used was designed for the DSM-IV, the only change in the diagnostic criteria for MD from DSM IV to 5 was that the latter removed the bereavement exclusion (29), and for this study we did not apply the bereavement exclusion in our scoring determination of lifetime MD. In addition to the DIS, participants were asked if they had any first-degree relatives with a history of depression (coded yes/no).

*Stress*. Two measures of stress exposure, were drawn from the PhenX Toolkit, a NIH-supported archive of psychosocial measures for use in genetics research (30). These measures included the Perceived Stress Scale (PSS) and a modified version of the Chronic Stress Scale (CSS) and were assessed at both baseline and 6-month follow-up interviews. The PSS consists of 10 items (Cronbach’s α=0.89) that assess general stress perceptions during the past 30 days, each on a five-point Likert scale ranging from *Never* to *Very Often* (31). The PSS does not ask about specific domains (i.e., items are asked about “difficult, stressful times” generally, and do not reference specific situations such as work, relationships, etc.). The PSS asks about the psychological *appraisal* of stressful situations (e.g., items ask about being unable to control important things, feeling overwhelmed, feeling nervous or “stressed”). In comparison, the CSS asks about *experiences* that many people find stressful across 11 life domains: general, finances, work, romantic relationships (partners, divorce/separated, non-partnered), social life, social isolation, family relationships/parenthood, residence/housing, and family health/caregiving using 47 items (see **Supplemental Table 2**). Items in the CSS are each measured on a three-point Likert scale in which each situation is rated as *Not true, Somewhat True*, or *Very True* for them at this time. However, respondents are not asked to rate their psychological appraisal of those situations (e.g., that is, they are not asked how stressed that situation makes them feel). The CSS generates both domain-specific and overall average scores, and for this analysis we used only the overall score (30).

*Demographic characteristics.* Demographic characteristics were assessed by self-report and included age (measured in years), sex (male, female), race (white, black), educational attainment (measured as highest schooling completed:<high school, high school, some college, college degree, and graduate/professional degree), annual household income (categorized as<$50,000, $50,000 to<$100,000, $100,000 to<$150,000 and ≥$150,000, based on the sample distribution), employment status (categorized as working full-time, working part-time, retired, and other), and marital status (categorized as married/cohabitating and not married (i.e., divorced/separated, widowed, or never married), based on the sample distribution).

### Statistical analysis

Descriptive characteristics were calculated using measures of central tendency, both for the sample overall and stratified by lifetime history of MD. Variation as a function of history of MD was evaluated using t-tests or Mann-Whitney U tests, as appropriate, for continuous variables and Fisher’s exact tests for categorical variables. Intraclass correlation coefficients (ICCs) were calculated using mixed models with random intercepts for sibship and for person-within-sibship. To compare the role of sibship-level and individual-level effects, we calculated two ICCs: ICC_sibship_ ( $\frac{\sigma_{sibship}^{2}}{\sigma_{person}^{2}+\sigma_{sibship}^{2}+\sigma_{residual}^{2}}$ ) and ICC_sibship+person_ ( $\frac{\sigma_{sibship}^{2}+\sigma_{person}^{2}}{\sigma_{person}^{2}+\sigma_{sibship}^{2}+\sigma_{residual}^{2}}$ ). Both ICCs were calculated using only sibships with complete data for both baseline and follow-up assessments. Values were log-transformed, as needed, to normalize distributions.

While this analysis is exploratory in nature, based on prior literature the overall expectations were that lifetime history of MD would be: 1) positively correlated with the measures of stress, 2) positively correlated with indicators of inflammation (e.g., higher IL-6, lower IL-1ra), and 3) positively correlated with indicators of diabetes risk (e.g., higher HbA1c). In addition, twins were expected to be highly correlated with each other on all these measures (both at baseline and over the 6-month follow-up period), but with lower correlations for psychosocial factors (e.g., stress scores) than biological factors (e.g., indicators of immune functioning), due to their unique environmental exposures but shared genetic background.

Analyses were performed in R (4.2.1) and SAS (9.4). R package lme4 (version 1.1-30) was used to calculate the ICCs. All p-values refer to two-tailed tests.

## Results

The mean age of participants at baseline was 52.9. The sample was predominantly female (68%), with a racial/ethnic composition of 77% white and 23% black. Using DSM-5 criteria, 33 individuals had a lifetime history of MD. There were 20 twin pairs discordant for having MD (19 of which were MZ), 7 pairs concordant for having MD (all MZ) and 17 pairs (16 of which were MZ) concordant for not having a history of MD. Examining recency, all episodes of MD last occurred at least 6 months prior to the baseline assessment, and >75% occurred at least a year prior to the baseline assessment, indicating that the MD cases identified here were recovered and not currently depressed.

**Table 1** shows descriptive statistics of participants both overall and stratified by lifetime history of MD. While differences were not statistically significant, participants with a history of MD were older, more likely to be female, white, and retired and had less education, lower household income, and were less likely to be married. Participants with a history of MD were about five times more likely to have a family history of MD (70% vs. 15%, p<.0001) and had higher diastolic (87.2 vs. 80.8 mmHg, p<0.002) and systolic (139.1 vs. 132.2 mmHg, p=0.05) BP. Waist circumference, BMI, CRP, HbA1c, blood glucose, and cortisol were comparable between the two groups, while IL-6 was higher among those with a history of MD (1.47 vs. 0.93 pg/mL, p=0.001).

**Table 1:** Baseline characteristics of the Mood and Immune Regulation in Twins Study.

	Overall (n=94)	Never MD (n=61)	Lifetime MD (n=33)	p-value
Demographic characteristics				
Age (mean, SD)	52.9 (7.8)	52.6 (7.5)	53.4 (8.4)	0.68
Female (N, %)	64 (0.68)	39 (0.64)	25 (0.76)	0.23
Confirmed zygosity (N, %)				0.86^F^
Monozygotic	85 (0.90)	54 (0.89)	31 (0.94)	
Dizygotic	4 (0.04)	3 (0.05)	1 (0.03)	
Singleton	5 (0.05)	4 (0.07)	1 (0.03)	
Race (N, %)				0.45^F^
Black	22 (0.23)	16 (0.26)	6 (0.18)	
White	72 (0.77)	45 (0.74)	27 (0.82)	
Education (N, %)				0.89^F^
<High school	4 (0.04)	2 (0.03)	2 (0.06)	
High school or equivalent	14 (0.15)	8 (0.13)	6 (0.18)	
Some college	29 (0.31)	20 (0.33)	9 (0.27)	
Completed college	29 (0.31)	19 (0.31)	10 (0.30)	
Graduate/professional school	18 (0.19)	12 (0.20)	6 (0.18)	
Household income (N, %)				0.83^F^
< $50,000	23 (0.24)	13 (0.21)	10 (0.30)	
$50,000-99,999	27 (0.29)	18 (0.30)	9 (0.27)	
$100,000-149,000	28 (0.30)	19 (0.31)	9 (0.27)	
≥$150,000	16 (0.17)	11 (0.18)	5 (0.15)	
Employment status (N, %)				0.11^F^
Employed full-time	68 (0.72)	42 (0.69)	26 (0.79)	
Employed part-time	12 (0.13)	11 (0.18)	1 (0.03)	
Retired	7 (0.07)	3 (0.05)	4 (0.12)	
Other	7 (0.07)	5 (0.08)	2 (0.06)	
Marital status (N, %)				0.64^F^
Married/cohabiting	66 (0.70)	44 (0.72)	22 (0.67)	
Divorced/separated, widowed or never married	28 (0.30)	17 (0.28)	11 (0.33)	
Family history of MD (N, %)	32 (0.34)	9 (0.15)	23 (0.70)	2.7e-07
Metabolic risk indicators				
Systolic BP [mmHg] (mean, SD)	134.6 (15.5)	132.2 (14.1)	139.1 (17.2)	0.05
Diastolic BP [mmHg] (mean, SD)	83.0 (8.5)	80.8 (6.8)	87.2 (9.9)	0.002
Waist circumference [cm] (mean, SD)	89.4 (13.6)	89.0 (13.3)	90.1 (14.3)	0.71
HbA1c [%] (mean, SD)	5.4 (0.6)	5.5 (0.7)	5.4 (0.4)	0.27
Random glucose [mg/dL] (mean, SD)	90.9 (13.7)	91.7 (15.5)	89.6 (9.8)	0.42
CRP [ng/mL] (median, IQR)	1383 (2482)	1383 (2766)	1440 (2141)	0.70^M^
IL-6 [pg/mL] (median, IQR)	1.10 (0.94)	0.93 (0.86)	1.47 (0.87)	0.001^M^
Cortisol [ng/mL] (median, IQR)	27.9 (21.7)	28.7 (27.5)	26.6 (14.6)	0.97^M^
BMI [kg/m^2^] (mean, SD)	28.1 (6.0)	27.9 (5.6)	28.6 (6.8)	0.60

MD, Lifetime history of major depression. All p-values are from t-tests except where otherwise indicated.

F, Fisher’s exact test; M, Mann-Whitney U Test.

**Table 2** and **Supplemental Figure 1** show the ICCs in metabolic risk, immune functioning, and stress indicators for individuals (self-over-time between baseline and 6-month follow-up), and for members of the sibling pair (between co-twins). For all measures, the ICC_sibship+person_ was higher than ICC_sibship_ as the result of the person-level random effects being nested within sibships. Height had a high ICC_sibship_ value and an ICC_sibship+person_ value that was not much larger than ICC_sibship_, indicating that similarity between an individual’s measurements with those of their co-twin was almost as high as similarity between an individual’s measurements over time. Other traits with high ICC_sibship_ such as waist circumference and BMI had higher values for ICC_sibship+person_, indicating that although there was high similarity between the twins there were also between-twin differences that remained stable across study visits. Notably, the PSS score and the two metabolic biomarkers (random glucose and HbA1c) had large differences between ICC_sibship_ and ICC_sibship+person_, suggesting that an individual’s measurements at baseline and 6-months were more similar to each other than to those of their co-twin. This indicates that these are measuring perceptions and biological states, respectively, that differ between the members of the pairs but which are relatively stable for individuals. Some immune indicators such as CRP had differences between the two ICCs that suggest persistent individual-specific differences, while others with a temporal rhythm (i.e., cortisol) or short-term fluctuations in response to environmental factors (i.e., IL-6) showed little increase in the ICC when including the random effect for person, suggesting that any between-twin differences may not be stable over time.

**Table 2:** Correlations in diabetes risk and stress measures among complete monozygotic twin pairs: Correlations of self-over-time and co-twin.

	N sibships	ICC_sibship+person_	ICC_sibship_
Clinical and Metabolic risk indicators			
Systolic blood pressure	37	0.733	0.544
Diastolic blood pressure	37	0.705	0.468
Height	37	0.974	0.957
Weight	36	0.989	0.783
BMI	36	0.984	0.752
Waist Circumference	29	0.977	0.763
Random glucose	35	0.795	0.244
HbA1c	37	0.879	0.294
Immune indicators			
Cortisol (log)	35	0.530	0.502
CRP (log)	35	0.770	0.394
IL-1RA (log)	36	0.844	0.547
IL-6 (log)	30	0.795	0.663
IL-10	24	0.790	0.638
TNF-Rii	36	0.679	0.608
Psychosocial stress indicators			
Perceived Stress Scale (PSS)	34	0.611	0.048
Chronic Stress Scale (CSS)	37	0.760	0.345

Intraclass correlation coefficients (ICCs) were calculated using mixed models with random intercepts for sibship and for person within sibship. Only MZ sibships in which both individuals participated at both timepoints were included.

**Figure 2:**
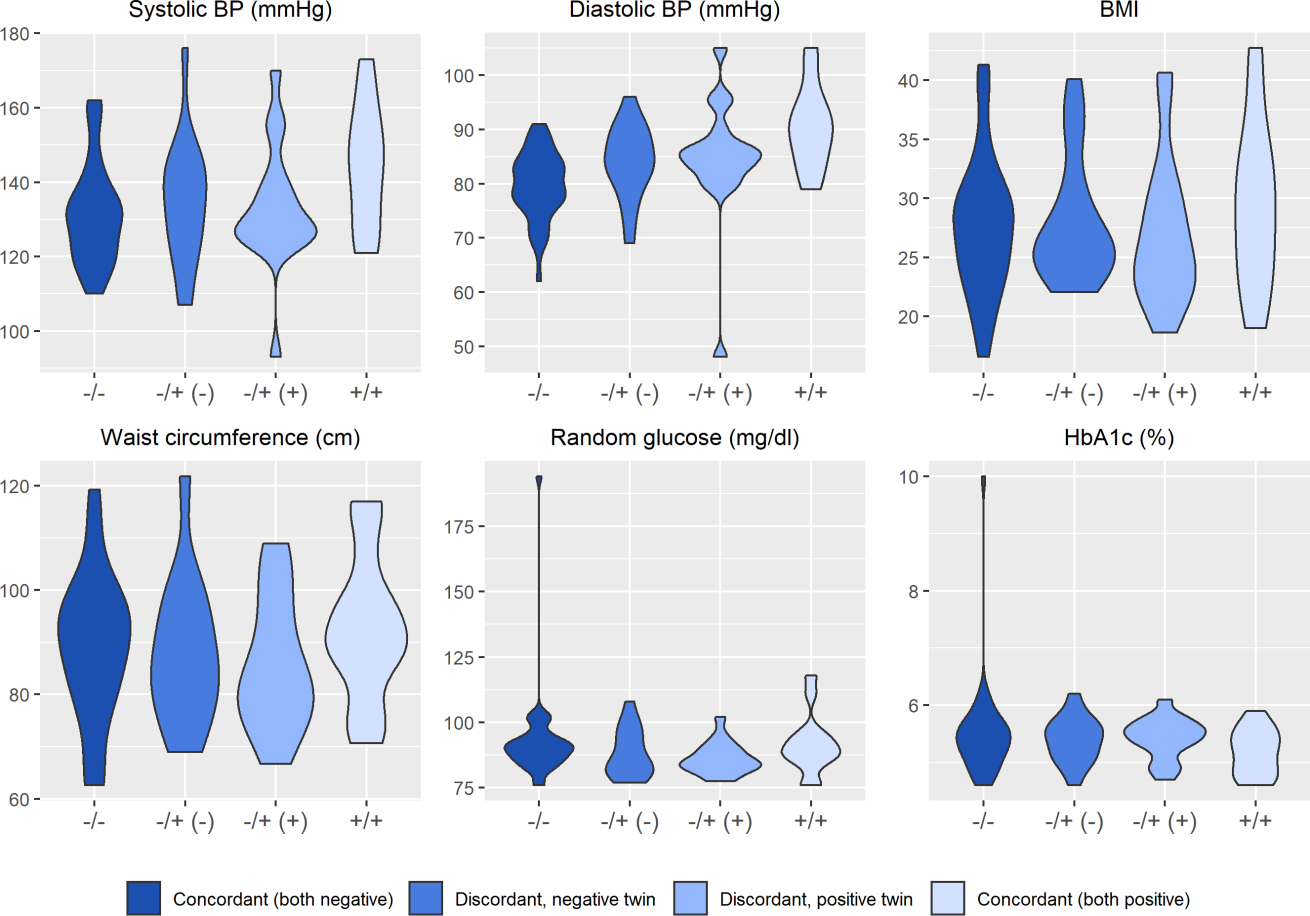
Distribution of clinical and metabolic markers arrayed by twin concordance for lifetime history of Major Depression (MD). Distribution of clinical and metabolic indicators by twin concordance for MD as assessed by the Diagnostic Interview Schedule. Units of the Y-axis for each indicator are shown in the subheading above each plot. Each plot is limited to the 42 twin pairs that were confirmed to be monozygotic using genetic testing. Sample size for each group shown in the legend: N_Corcordant (both do not have MD)_ = 16 twin pairs (32 individuals), N_Discordant for MD (-)_ = 19 twin pairs (19 individuals), N_Discordant for MD (+)_ = 19 twin pairs (19 individuals), and N_Corcordant (both have MD)_ = 7 twin pairs (14 individuals).

**Figure 3:**
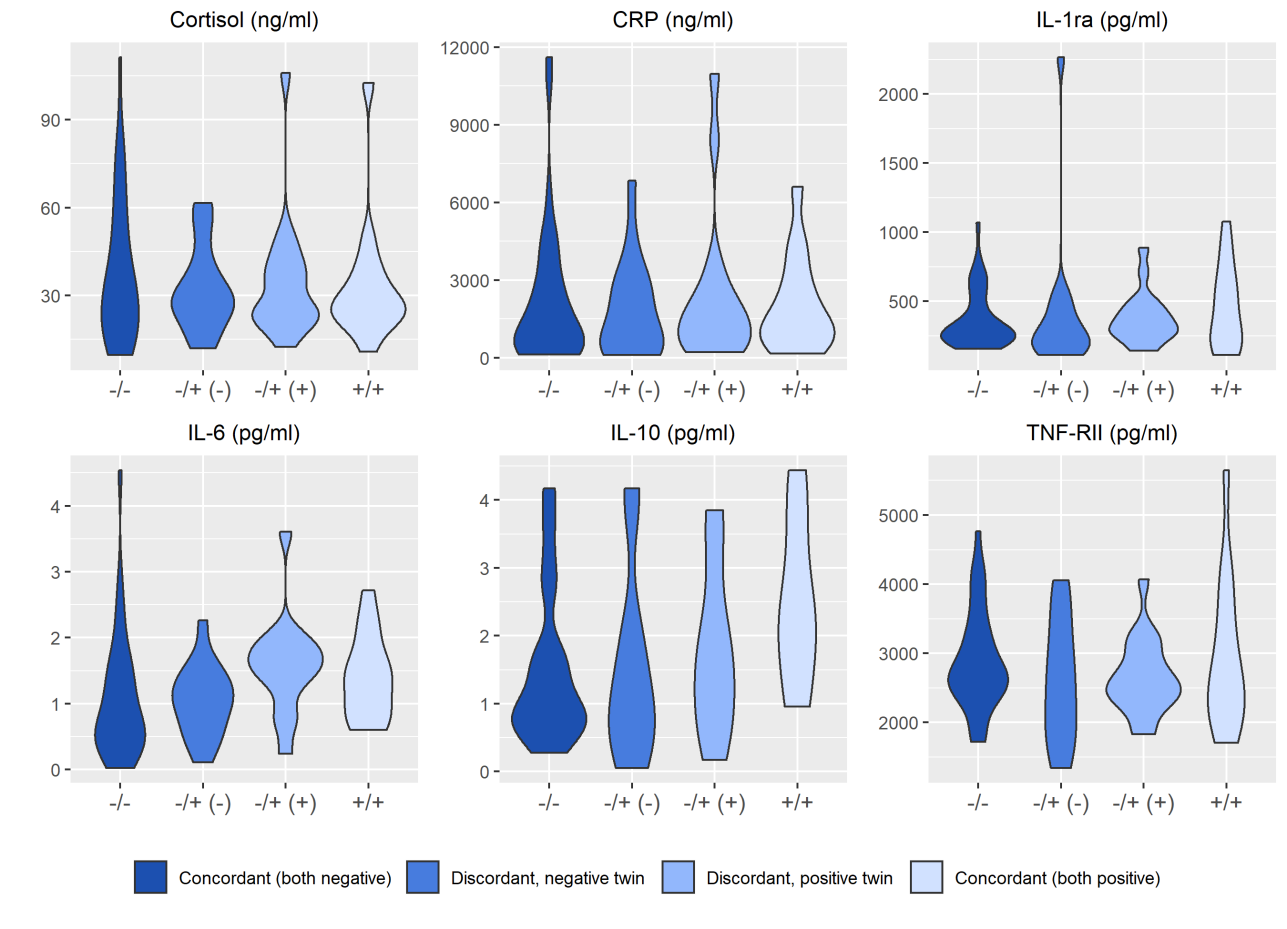
Distribution of immune markers arrayed by twin concordance for lifetime history of Major Depression (MD). Distribution of immune indicators by twin concordance for MD as assessed by the Diagnostic Interview Schedule. Units of the Y-axis for each indicator are shown in the subheading above each plot. Each plot is limited to the 42 twin pairs that were confirmed to be monozygotic using genetic testing. Sample size for each group shown in the legend: N_Corcordant (both do not have MD)_ = 16 twin pairs (32 individuals), N_Discordant for MD (-)_ = 19 twin pairs (19 individuals), N_Discordant for MD (+)_ = 19 twin pairs (19 individuals), and N_Corcordant (both have MD)_ = 7 twin pairs (14 individuals).

**Figure 4:**
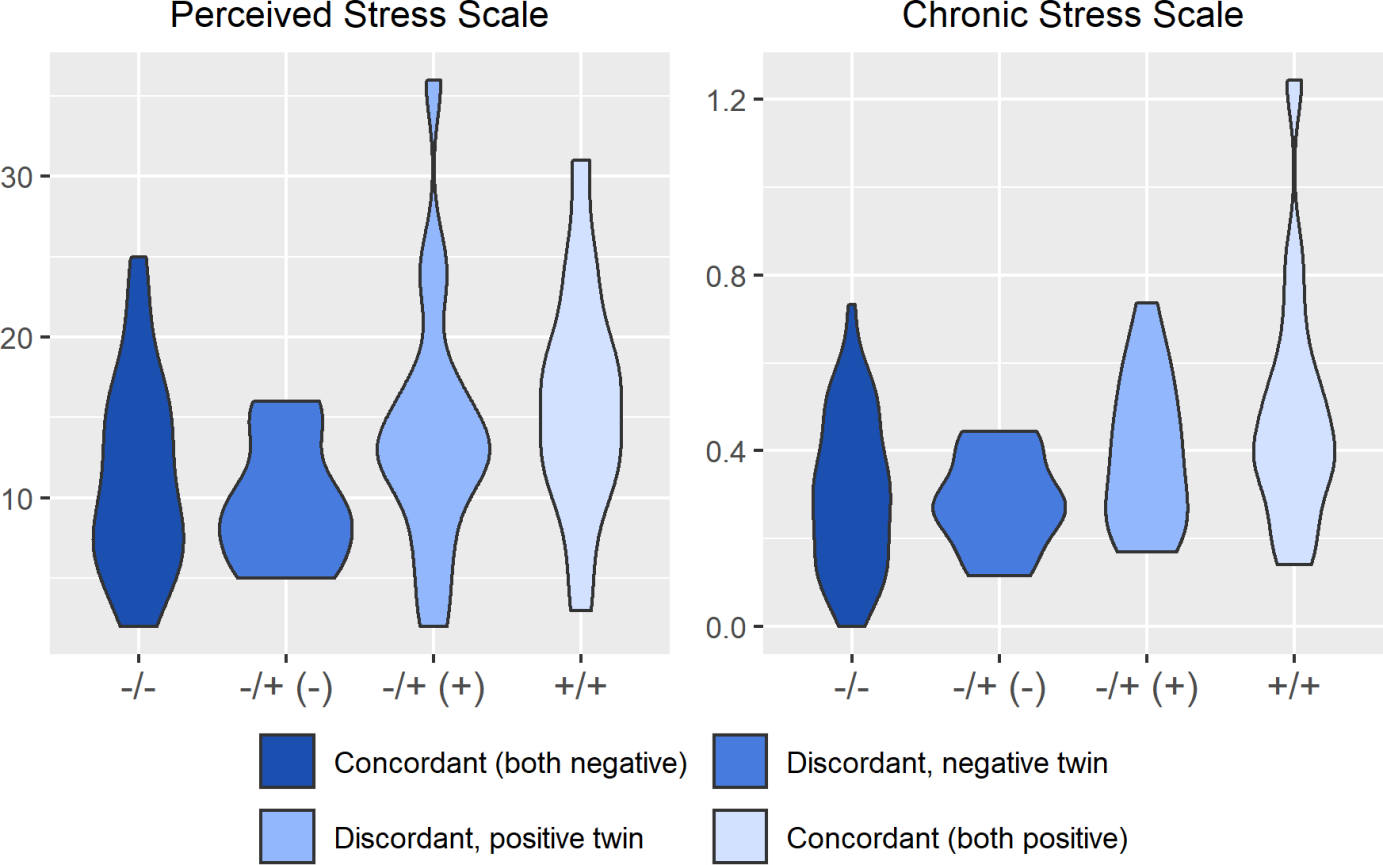
Distribution of psychosocial stress arrayed by twin concordance for lifetime history of Major Depression (MD). Distribution of stress indicators (Perceived Stress Scale and Chronic Stress Scale) by twin concordance for MD as assessed by the Diagnostic Interview Schedule. Each plot is limited to the 42 twin pairs that were confirmed to be monozygotic using genetic testing. Sample size for each group shown in the legend: N_Corcordant (both do not have MD)_ = 16 twin pairs (32 individuals), N_Discordant for MD (-)_ = 19 twin pairs (19 individuals), N_Discordant for MD (+)_ = 19 twin pairs (19 individuals), and N_Corcordant (both have MD)_ = 7 twin pairs (14 individuals).

**Figures 2** **–** **4** display an array of violin plots showing the distribution of the metabolic, immune, and psychosocial indicators for the 42 pairs of monozygotic twins by MD status of the pair (concordant for not having MD, discordant for MD, and concordant for having MD). Examining only the values for the discordant pairs (which by the nature of the co-twin design represent comparisons matched on key confounders such as age, sex, race, socioeconomic status in childhood, and genetic liability), while there is relatively little difference in the metabolic (**Figure 2**) and most immune indicators (**Figure 3**), the twin with MD tended to have higher levels of IL-6 and reported more stress on both the PSS and CSS scales (**Figure 4**). The direction of these differences was consistent with that shown by twins from pairs concordant for having MD.

## Discussion

The debate juxtaposing ‘nature *versus* nurture’ has long been set aside, as it is established that both genetic liability and environmental exposures are fundamental determinants of health conditions, including depression and diabetes (4). However, studies that incorporate a wide range of genetic, epigenetic, and environmental data to investigate how these mechanisms inter-relate to shape mental and physical health have only recently emerged (32). Beginning with the biopsychosocial model as a conceptual foundation, this paper described the study design, data collection protocols, sample characteristics, and initial findings from a longitudinal co-twin study. While other studies have examined differences in gene expression between MZ twins at a single point in time (33, 34), to our knowledge this is one of the few studies (35, 36) with longitudinal data on gene expression within a co-twin design.

While exploratory in nature, the findings from this study emphasize the need to consider both genetic and environmental factors for MD and diabetes. In the sample overall, MD was associated with higher IL-6 and blood pressure, but not other indicators of diabetes risk. Comparing members of discordant pairs, the twin with MD reported more stress than their co-twin in line with our expectations; however, differences in metabolic and immune indicators comparing the discordant pairs were more modest. As stated above, the MIRT study can be conceptualized as a two-by-two study design, in which individuals can be compared to both their co-twin at baseline and to themselves at the 6-month follow-up. This comparison was quantified by the ICC analysis, which clarified that variation in some risk factors (e.g., BMI) is primarily due to factors shared among both members of each sibships (i.e., genotype and early-life environmental factors), while variation in other risk factors (e.g., perceived stress) is primarily reflective of an individual’s unique environmental exposures which also have a degree of stability (at least over a 6-month period). Taken together, this analysis helps clarify which psychosocial characteristics are potentially more modifiable and thus could be prioritized as targets for intervention (20).

### A need for both breadth and depth of genetically-informative studies

The past few decades have yielded tremendous advances in genetic epidemiology, driven by large repositories such as the UK Biobank (37) and consortia that aggregate multiple samples (e.g., Psychiatric Genetics Consortium, Diabetes Genetics Replication and Meta-analysis) (38). These datasets have been vital to the advancement of health research, but they are not without their limitations. First, while greater sample size clearly improves the precision of effect estimates, this does not necessarily result in more valid inferences; this is particularly relevant for mental health phenotypes that require extensive interviews for appropriate case identification (39), which is often not possible in large samples. Second, so-called “big data” can lead to complacency with statistically significant but scientifically trivial findings because sample size has an inverse relationship with detectable effect size; therefore, as sample size increases, it is logical to decrease alpha or commit to a more ambitious alternative hypothesis (40). Yet in practice we tend to apply the same arbitrary alpha value for a wide range of sample sizes and tout any p-value less than 0.05 as noteworthy.

Finally, large repositories and aggregated samples often lack “depth.” Depth in data here refers to both comprehensive measurement of exposures and outcomes, as well as consideration of study design and data collection protocols. Depth can also be characterized by situating data within a theoretical context that considers how genetic factors intersect with psychological, social, and behavioral pathways over the life course. The MIRT data includes detailed questionnaires about mental health, stress, coping, and health behaviors, often using multiple instruments and measuring different but related constructs, allowing nuanced examination of how such factors inter-relate and influence health. In sum, there is both a need for “breadth” and “depth” in genetically-informative study designs, and MIRT serves as an example of the latter.

### Strengths and limitations

Strengths of this study include the racial diversity of the sample (e.g., African Americans are significantly under-represented in health research) (41), multiple assessments of key exposures (e.g., including both the PSS and CSS) which permits examination of the role of measurement error on our inferences, the longitudinal study design, and the use of appropriate tissue (e.g., white blood cells) for examining immune-related RNA mechanisms. Limitations include the limited sample size and relatively brief follow-up period, both of which increase type 2 error. Therefore, the results of MIRT should be considered primarily as exploratory and hypothesis-generating. Due to the lengthy DIS protocol we also did not re-assess MD at the 6-month follow-up to mitigate respondent burden, and therefore it is possible that incident or recurrent episodes of depression occurred between these assessments that were not captured by our study. If the biological mechanisms linking psychosocial factors to health are general rather than specific in nature (e.g., not MD per say, but rather a result of any form of persistent psychological distress), these processes may be captured by measures such as the PSS which assess current exposures. All cases of MD were resolved (i.e., last occurred at least 6 months before baseline) and this may have reduced our ability to detect associations between MD and the biological measures, especially if these relationships are primarily acute in nature. However, lifetime history of MD was positively associated with scores on the PSS and CSS, suggesting that while these participants did not meet DSM diagnostic criteria at the time, they were still experiencing higher levels of psychosocial distress than those who had never had MD.

### Conclusions and plans for future research

As a longitudinal study of twins, MIRT is useful for exploring relationships among biological, psychosocial and behavioral indicators of health. Because MZ twins are matched on an array of confounders such as age, sex, race, childhood socioeconomic status, and genetic liability, the effects of individual-specific environmental exposures can be inferred with more certainty. This study collected a broad range of psychological, social, and biological measures, and thus it has the potential for exploring an array of scientific questions derived from the biopsychosocial model (2). Future analyses of the MIRT cohort will explore how MD relates to changes in physiologic and psychological indicators over time, as well as within-pair differences in gene expression. Such decompositions of the role of genetic and environmental sources of depression and diabetes risk are only possible because of the genetically-informed nature of the MIRT sample. It is hoped that this study will eventually add to the growing body of literature on the complex biological and epigenetic processes that contribute to the connections between psychosocial factors and health (42).

## Data availability statement

Participant recruitment, data collection materials, and data codebooks are publicly available at the Open Science Framework (https://osf.io/24vpb/). The datasets generated by the MIRT Study are available by contacting the corresponding author. Sequencing data is not publicly available due to concerns regarding participant/patient anonymity. Requests to access the datasets should be directed to the corresponding author.

## Ethics statement

The studies involving human participants were reviewed and approved by Institutional Review Board at Virginia Commonwealth University (Protocol ID: HM15108). The patients/participants provided their written informed consent to participate in this study.

## Author contributions

BM conceptualized the study, obtained funding for the data collection, and wrote the first draft of this manuscript. KK conducted the statistical analysis and assisted with drafting the manuscript. EB conducted the literature review and assisted with drafting the manuscript. JC led the data collection effort and assisted with drafting the manuscript. All authors contributed to the article and approved the submitted version.
