# Supplementary material for: Leveraging a genetically-informative study design to explore depression as a risk factor for type 2 diabetes: Rationale and participant characteristics of the Mood and Immune Regulation in Twins Study
Source: Front Clin Diabetes Healthc. 2023 Mar 17;4:1026402. doi: 10.3389/fcdhc.2023.1026402 (PMC10064086; doi:10.3389/fcdhc.2023.1026402)

**Supplementary materials**

1. Repository of data collection materials and codebooks for the MIRT Study.
2. Sensitivity and coefficients of variation for immune biomarker assays.
3. Description of the modified Chronic Stress Scale used in the MIRT Study
4. Plot of correlations between-sibships vs. within-person-over-time for metabolic, immune and psychosocial indicators

**Repository of data collection materials**

Participant recruitment, data collection materials, and data codebooks are publicly available at the Open Science Framework (<https://osf.io/24vpb/>).

Citation for this repository: Mezuk, B., & Johns, L. (2022, August 18). Leveraging genetically-informative study designs to explore the relationship between depression and type 2 diabetes: Rationale and baseline characteristics of the Mood and Immune Regulation in Twins Study. Retrieved from osf.io/24vpb

**Supplemental Table 1**: Sensitivity and coefficients of variation (CV) for immune biomarker assays in the Mood and Immune Regulation in Twins Study

| **Test** | **Minimum detectable value** | **Intra-Assay CV** | **Inter-Assay CV** |
| --- | --- | --- | --- |
| TNF-RII^1^ | 0.6 pg/ml (0.2-2.3) | 2.6 - 4.8% | 3.5 - 5.1% |
| CRP^2^ | 0.010 ng/ml (0.005-0.022) | 3.8 - 8.3% | 6.0 - 7.0% |
| Cortisol^3^ | 0.071 ng/ml (0.030-0.111) | 6.3 - 9.2% | 9.3 - 21.2% |
| IL-1ra^4^ | 6.3 pg/ml (2.2-18.3) | 3.7 - 7.3% | 6.7 - 11.0% |
| IL-6^5^ | 0.039 pg/ml (0.016-0.110) | 6.9 - 7.8% | 6.5 - 9.6% |
| IL-10^6^ | 0.09 pg/ml (0.03-0.17) | 4.6 - 9.3% | 7.8 - 13.1% |
| ^1^ R&D Systems Quantikine ELISA Human TNF-RII/TNFRSF1B.  ^2^ R&D Systems Quantikine Human CRP Immunoassay.  ^3^ R&D Systems Parameter Cortisol.  ^4^ R&D Systems Quantikine ELISA Human IL-1ra/IL-1F3.  ^5^ R&D systems Quantikine HS ELISA Human IL-6.  ^6^ R&D systems Quantikine HS Human IL-10.  *Note*: No maximum detectable limits were specified in the manufacturer documentation | | | |

**Supplemental Table 2**. Comparison of Original and Modified Items of the Chronic Stress Scale used in the Mood and Immune Regulation in Twins Study

| Domain | No. | Original item wording | No. | Wording used in MIRT |
| --- | --- | --- | --- | --- |
| General | 1 | You are trying to take on too many things at once. | 1 | Identical to original |
|  | 2 | There is too much pressure on you to be like other people. | 2 | Identical to original |
|  | 3 | Too much is expected of you by others. | 3 | Identical to original |
| Money and Finance | 4 | You don’t have enough money to buy the things you or your **kids** need. | 4 | You don’t have enough money to buy the things you or your **family** need. |
|  | 5 | You have a long-term debt or loan. | 5 | Identical to original |
|  | 6 | Your rent or mortgage is too much. | 6 | Identical to original |
|  | 7 | You don't have enough money to take vacations. | 7 | Identical to original |
|  | 8 | You don’t have enough money to make a down payment on a home. | 8 | Identical to original |
| Work | 9 | You have more work to do than most people. | 9 | Identical to original |
|  | 10 | Your supervisor is always monitoring what you do at work. | 10 | Identical to original |
|  | 11 | You want to change jobs or career but don't feel you can. | 11 | Identical to original |
|  | 12 | Your job often leaves you feeling both mentally and physically tired. | 12 | Identical to original |
|  | 13 | You want to achieve more at work but things get in the way. | 13 | Identical to original |
|  | 14 | You don't get paid enough for what you do. | 14 | Identical to original |
|  | 15 | Your work is boring and repetitive. | 15 | Identical to original |
|  | 16 | You are looking for a job and can’t find the one you want. |  | Not asked in MIRT |
| Romantic Relationships (for those with a current partner) | 17 | You have a lot of conflict with your partner. | 16 | Identical to original |
|  | 18 | Your relationship restricts your freedom. | 17 | Identical to original |
|  | 19 | Your partner doesn't understand you. | 18 | Identical to original |
|  | 20 | Your partner expects too much of you. | 19 | Identical to original |
|  | 21 | You don’t get what you deserve out of your relationship. | 20 | Identical to original |
|  | 22 | Your partner doesn’t show enough affection. | 21 | Identical to original |
|  | 23 | Your partner is not committed enough to your relationship. | 22 | Identical to original |
|  | 24 | Your sexual needs are not fulfilled by this relationship. |  | Not asked in MIRT |
|  | 25 | Your partner is always threatening to leave or end the relationship | 23 | Identical to original |
| Romantic Relationships (for those without a current partner) | 26 | You wonder whether you will ever get married | 24 | Identical to original |
|  | 27 | You find it is too difficult to find someone compatible with you. | 25 | Identical to original |
| Romantic Relationships (for those with a former partner) | 28 | You have a lot of conflict with your ex-spouse. | 26 | Identical to original |
|  | 29 | You don't see your children from a former relationship as much as you would like. | 27 | Identical to original |
| Isolation | 30 | You are alone too much. | 28 | Identical to original |
| Parenthood | 31 | You wish you could have children but you cannot. |  | Not asked in MIRT |
|  | 32 | One of your children seems very unhappy. | 29 | Identical to original |
|  | 33 | You feel your children don't listen to you. | 30 | Identical to original |
|  | 34 | A child’s behavior is a source of serious concern to you. | 31 | **Your children’s** behavior is a source of serious concern to you. |
|  | 35 | One or more children do not do well enough at school or work. | 32 | Identical to original |
|  | 36 | Your children don't help around the house. | 33 | Identical to original |
|  | 37 | One of your children spends too much time away from the house. | 34 | **Your children** spend too much time away from the house. |
|  | 38 | You feel like being a housewife is not appreciated. |  | Not asked in MIRT. |
| Social Life | 39 | You have to go to social events alone and you don’t want to. | 35 | Identical to original |
|  | 40 | Your friends are a bad influence | 36 | Identical to original |
|  | 41 | You don’t have enough friends | 37 | Identical to original |
|  | 42 | You don’t have time for your favorite leisure time activities | 38 | Identical to original |
| Residence | 43 | You want to live farther away from your family. | 39 | Identical to original |
|  | 44 | You would like to move but you cannot. | 40 | Identical to original |
|  | 45 | The place you live is too noisy or too polluted. | 41 | Identical to original |
|  | 46 | Your family lives too far away. | 42 | Identical to original |
| Family | 47 | Someone in your family or a close friend has a long-term illness or handicap. | 43 | Someone in your family or a close friend has a long-term illness or **disability**. |
|  | 48 | You have a parent, a child, or a spouse or partner who is in very bad health. | 44 | Identical to original |
|  | 49 | Someone in your family has an alcohol or drug problem. | 45 | Identical to original |
|  | 50 | A long-term health problem prevents you from doing the thigs you like to do. | 46 | Identical to original |
|  | 51 | You take care of an aging parent almost every day. | 47 | You take care of an aging parent **or relative** almost every day. |
| Response options for all items were *Not true, somewhat true,* or *Very true* *at this time*, which is identical to the original scoring. | | | | |

**Supplemental Figure 1**: Plot of ICC values of within pair (sibship) vs. within person (over time) of metabolic, immune, and psychosocial indicators


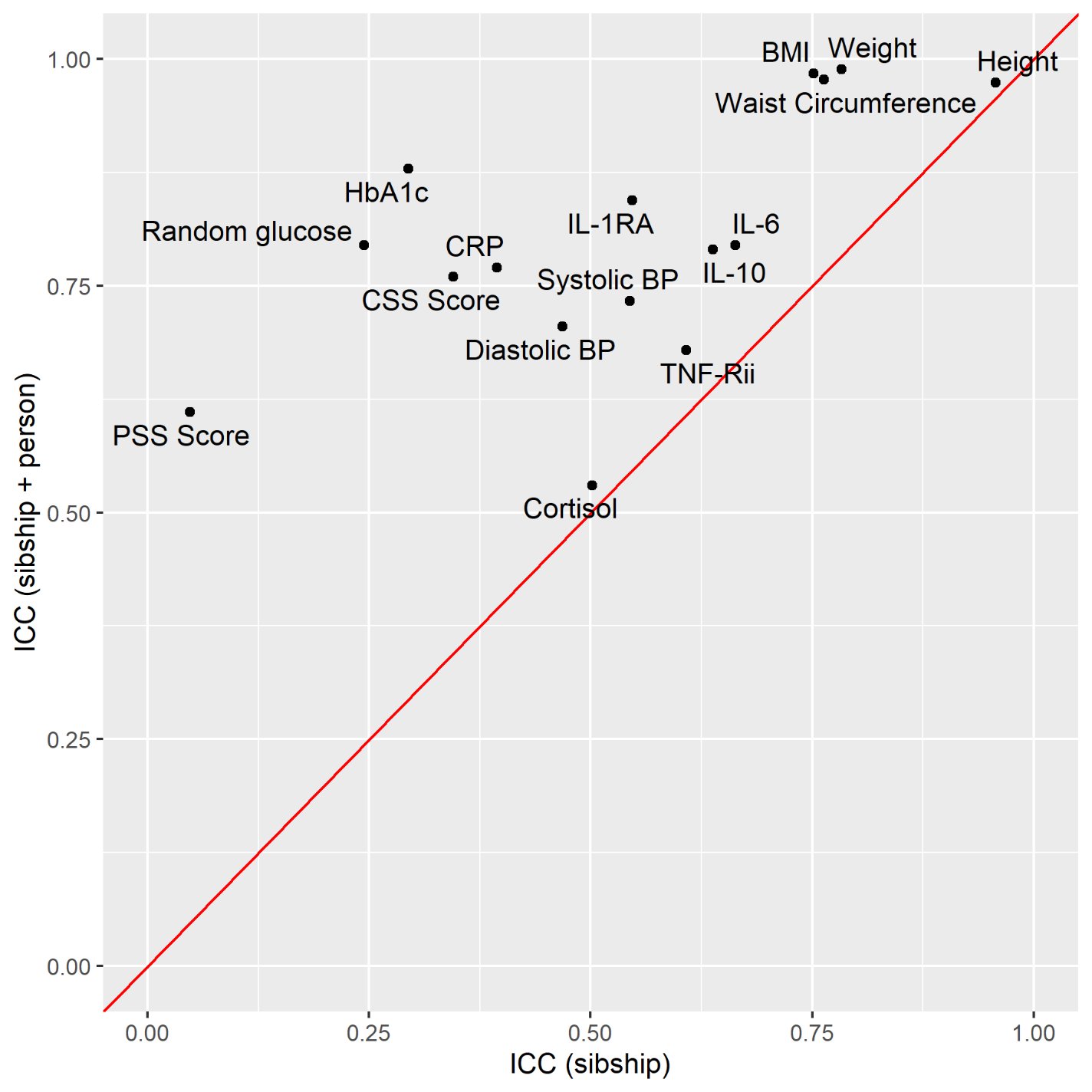

Supplement: Supplementary file 1 [file Table_1.docx]
